# Multidimensional Dynamics of the Proteome in the Neurodegenerative and Aging Mammalian Brain

**DOI:** 10.1016/j.mcpro.2021.100192

**Published:** 2021-12-31

**Authors:** Byron Andrews, Alan E. Murphy, Michele Stofella, Sarah Maslen, Leonardo Almeida-Souza, J. Mark Skehel, Nathan G. Skene, Frank Sobott, René A.W. Frank

**Affiliations:** 1 MRC Laboratory of Molecular Biology, Cambridge, UK; 2 UK Dementia Research Institute at Imperial College London, London, UK; 3 Department of Brain Sciences, Imperial College London, London, UK; 4 Astbury Centre of Molecular Structural Biology, Faculty of Biological Sciences, University of Leeds, Leeds, UK; 5 Helsinki Institute of Life Science - HiLIFE, Institute of Biotechnology and Faculty of Biological and Environmental Sciences, University of Helsinki, Helsinki, Finland

**Keywords:** protein turnover, SILAM, Alzheimer's disease, proteomics, neurodegeneration, aging, AD, Alzheimer’s disease, DEG, differentially expressed gene, EWCE, expression weighted cell type enrichment

## Abstract

The amount of any given protein in the brain is determined by the rates of its synthesis and destruction, which are regulated by different cellular mechanisms. Here, we combine metabolic labeling in live mice with global proteomic profiling to simultaneously quantify both the flux and amount of proteins in mouse models of neurodegeneration. In multiple models, protein turnover increases were associated with increasing pathology. This method distinguishes changes in protein expression mediated by synthesis from those mediated by degradation. In the *App*^*NL-F*^ knockin mouse model of Alzheimer’s disease, increased turnover resulted from imbalances in both synthesis and degradation, converging on proteins associated with synaptic vesicle recycling (Dnm1, Cltc, Rims1) and mitochondria (Fis1, Ndufv1). In contrast to disease models, aging in wild-type mice caused a widespread decrease in protein recycling associated with a decrease in autophagic flux. Overall, this simple multidimensional approach enables a comprehensive mapping of proteome dynamics and identifies affected proteins in mouse models of disease and other live animal test settings.

## Highlights

• Multidimensional proteomic screen to detect imbalances in mouse models of disease.
• Increased proteome turnover in multiple symptomatic neurodegeneration mouse models.
• Healthy aging is associated with a global decrease in protein turnover.

## Graphical Abstract

**Figure:**
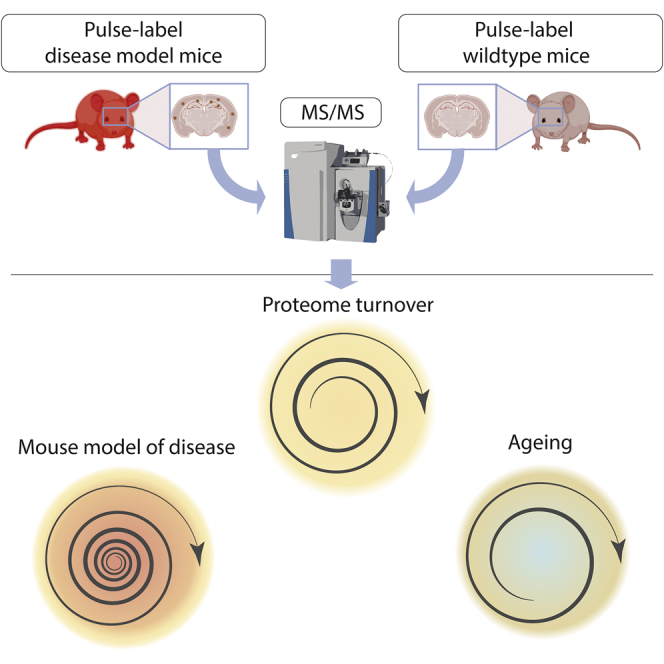

## In Brief

Neurodegenerative diseases are characterized by the abnormal accumulation of aggregated proteins in the brain. Using *in vivo* pulse isotope labeling, we screened the proteome for changes in protein turnover and abundance in multiple mouse models of neurodegeneration. These data suggest that the disease state of pathologically affected tissue is characterized by a proteome-wide increase in protein turnover and repair. In contrast, in healthy wild-type mice, aging in the mammalian brain is associated with a global slowdown in protein turnover.

In the mammalian brain, the rate of protein turnover typically ranges from minutes to several days and is determined by both the rate of synthesis and degradation (1, 2, 3, 4, 5). This extraordinary flux poses a particular challenge for the brain because information must outlive the molecular substrates in which they are stored (6, 7) but is a necessary protein repair mechanism to counter the accumulation of damaged proteins (8, 9). In the adult brain, almost all neurons are terminally differentiated and most neuronal protein repair is not helped by mitotic cellular turnover, as applies in many other tissues. The rate of proteome turnover is regulated by multiple factors and mechanisms, including ubiquitin-proteasome and autophagy-mediated degradation (10, 11, 12). Protein turnover perturbations cause severe neurological dysfunction (13). Indeed, the most common neurodegenerative diseases are characterized by imbalances in the turnover of a few proteins, resulting in their accumulation into misfolded protein aggregates (14, 15). These inclusions appear to be resistant to cellular mechanisms of protein repair (16). Abnormal inclusions in nonneuronal cell culture have been shown to have widespread impact on the proteome and its functions (17, 18, 19). However, it is not known if neurodegenerative diseases have an impact on global proteome turnover in the mammalian brain.

Neurodegenerative diseases are characterized by synapse loss, cognitive decline, and eventual neuronal death. What triggers these diseases is unknown, except for a very small subset that is caused by familial mutations (20, 21). Yet, even in these rare cases, a comprehensive understanding of what downstream pathological pathways are involved in cognitive decline, synapse, and neuronal loss is lacking (22).

In most neurodegenerative diseases, including Alzheimer’s disease (AD), it is apparent that pathology arises over many years, perhaps decades (15, 23, 24). Consequently, pathology in the early stages of the disease could be masked by increased repair and adaptation (25, 26, 27, 28). Therefore, methods capable of detecting these changes in repair could indicate the earliest upstream pathways of the disease (22).

To study AD, mouse models provide an excellent setting because genetic approaches can be applied within an organism that is neuroanatomically and molecularly similar to humans (29). Many useful models are available that show varying signs of cognitive decline and synaptic loss, but none reflect the full cascade of pathology including neuronal death (30). Thus, approaches are required that can reconcile the range of molecular abnormalities in different mouse models of disease and identify affected molecular pathways.

*In vivo* metabolic labeling and global proteomic profiling have the capacity to measure the dynamics of individual proteins throughout the proteome (31, 32, 33, 34, 35). Measuring precisely the absolute turnover rates of individual proteins *in vivo* is important, though challenging, because of the diverse routes of metabolite incorporation and recycling (9, 36). In contrast, measuring the relative change in protein turnover in disease models compared with control animals could enable the identification of pathways altered in the disease state. Here, we first established a method using ^13^C heavy lysine (K6) labeling to detect global proteome turnover change in mice. Next, we developed the assay to simultaneously measure changes in protein turnover and abundance *in vivo*. This multiplex screen of proteome dynamics is applicable to any protein in any tissue and distinguishes between changes driven by synthesis or degradation of a protein. We applied this screen to quantify ∼1000 proteins in three mouse models of neurodegenerative disease at presymptomatic and symptomatic ages. In all models that we tested, increased neuropathology is associated with increased protein turnover and changes in the amount of some specific proteins, caused by measurable alterations in their synthesis or degradation. Finally, we used the method to investigate the proteome dynamics that are associated with aging in healthy mice. Global protein turnover decreased with age, which was associated with a slowdown in autophagy. This resource reveals novel signatures of pathology, facilitates comparisons between different mouse models of disease, and contrasts neurodegeneration with the mechanisms of aging.

## Experimental Procedures

### Experimental Design and Statistical Rationale

A sample size of three mice in each cohort was used for each experiment. Disease model and control cohorts in each experiment consisted of a disease model and age-matched controls. Disease and control mice were bred from founder mice, ensuring a highly similar genetic background. Five independent experiments were performed testing (i) 113 days postnatal age (P113) *TgCRND8* (37), (ii) P285 *TgCRND8*, (iii) P186 *App*^*NL-F/NL-F*^ knockin (38), (iv) P548 *App*^*NL-F/NL-F*^ knockin, and (v) P120 *SOD1-G93A* mice modeled ALS (39). The change in the mean global proteome turnover in mouse models of disease was compared with wild-type with a two-sided unpaired Student’s *t* test and considered significant below a threshold *p* < 0.05.

### Metabolic Labeling of Live Mice

Animals were treated in accordance with UK Animal Scientific Procedures Act (1986) and NIH guidelines. All animal experiments were approved by the MRC Laboratory of Molecular Biology AWERB (Animal Welfare and Ethical Review Board). Mouse genotyping was performed by PCR using primers specific for the mutant allele on every mouse. Mouse proteins were globally labeled ^13^C heavy lysine (Lysine-6; K6) by feeding with mice Lys-6 food (Silantes) for 6 to 8 days. All transgenic models of disease were fed K6 food for 6 days, as were the control mice used in the aging analysis. To counteract the decline in protein turnover that is observed in age, the 18-month-old *App*^*NL-F/NL-F*^ and control of mice were fed for 8 days. Mice were kept in cages separated by genotype and labeled in groups of six: three control and three experimental mice. The mass of each mouse and food consumed were recorded throughout the experiments, and the primary control for assessing the level of heavy label incorporation was the measurement of plasma protein turnover.

**Fig. 1:**
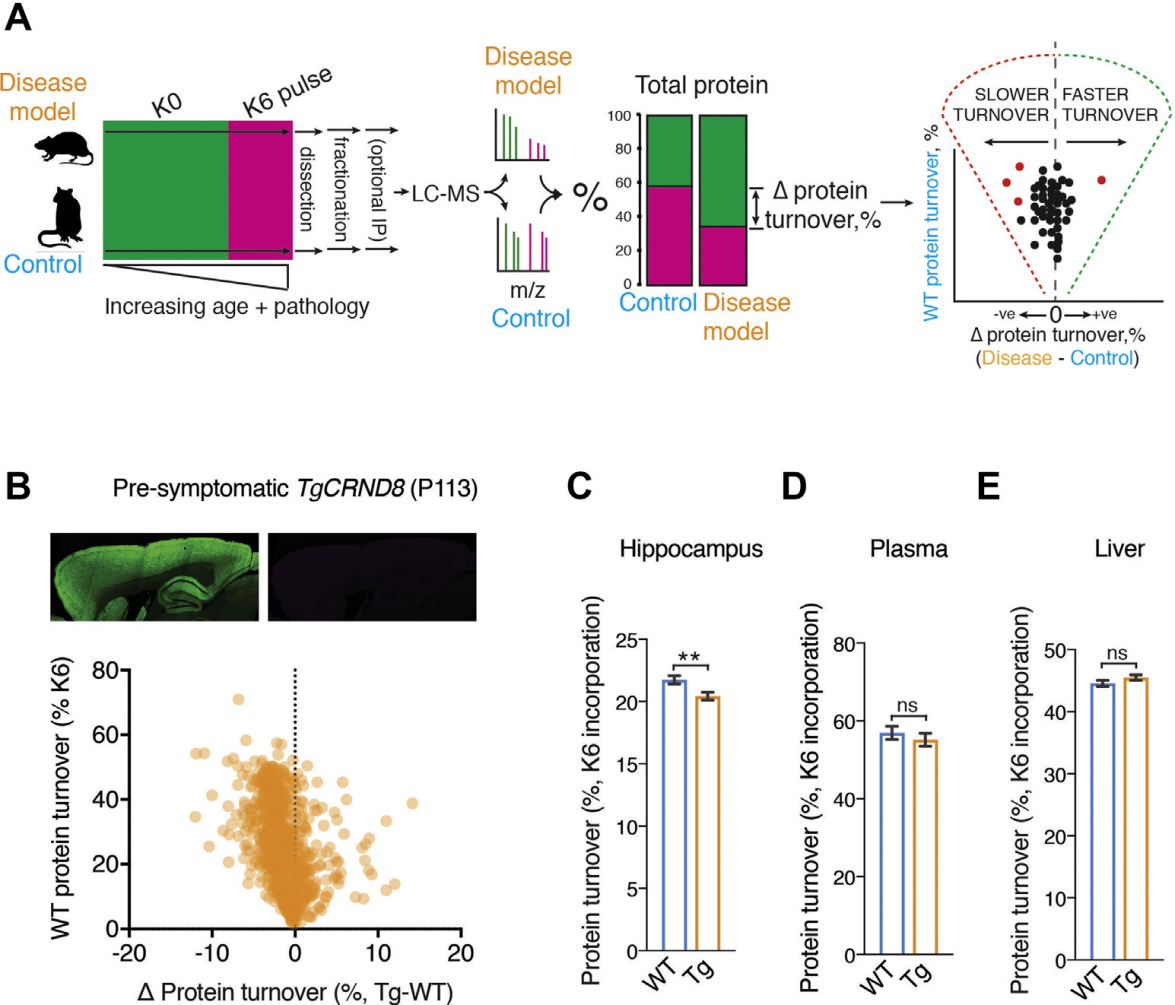
**Metabolic labeling of live mice to measure changes in protein turnover.***A*, *left*, schematic summarizing the ^13^C heavy lysine (K6) labeling method for measuring changes in protein turnover in mice. Cohorts of genetically and age-matched mice were maintained on regular food until the desired labeling window. The groups of mice are then switched to K6 diet for an identical period before tissues were processed for orbitrap LC-MS/MS. *Middle*, ion pairs with a 6 Da difference in mass were detected and sequenced, corresponding to peptides with and without K6. The relative incorporation of K6 was calculated in disease and wild-type mice. *Right*, the mean difference in K6 incorporation is calculated for each protein and plotted (*x*-axis) to highlight slowdown or increases in protein turnover *versus* whether a protein has fast or slow turnover (WT K6 incorporation, *y*-axis). *B*, *top*, immunohistochemical detection of synaptic marker, *left*, Psd95 and *right*, β-amyloid pathology in sagittal sections of presymptomatic *TgCRND8* (P113) mouse brain. *Bottom*, scatter plot showing protein turnover changes in the hippocampus of presymptomatic (P113) *TgCRND8* mice (see supplemental Table S1). In total, 1392 proteins were quantified in both diseased and healthy cohorts of mice (2–3 mice in each cohort). The mean difference of K6 incorporation for each protein (*x*-axis, Tg - WT) was plotted against the incorporation in WT (*y*-axis). Three *TgCRND8* and three age-, sex-, and background-matched WT mice were K6 labeled for 6 days. These data indicate a global decrease in protein turnover for hippocampal proteins with both a slow and fast turnover. *C*, bar chart showing average protein turnover of 1392 proteins in the hippocampus of *TgCRND8* and WT mice. An overall 6.1% slowdown in turnover was recorded in the hippocampus (*p* = 0.0037). Error bars indicate SEM. ∗∗*p* < 0.01. *D*, bar chart showing average plasma protein turnover in *TgCRND8* and WT mice. No significant difference was detected (*p* = 0.128, n = 118). Error bars indicate SEM. ns, not significant. *E*, bar chart showing average liver protein turnover in *TgCRND8* and WT mice. No significant difference was detected (*p* = 0.17, n = 590). ns, not significant.

**Fig. 2:**
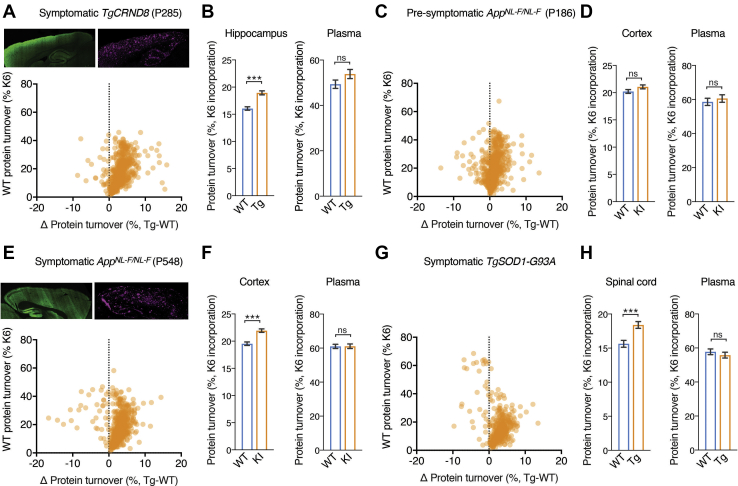
**Differences in protein turnover in transgenic and knock-in models of AD and a transgenic model of ALS.***A*, *top*, immunohistochemical detection of synaptic marker, *left*, Psd95 and *right*, β-amyloid pathology in sagittal sections of symptomatic *TgCRND8* (P285) mouse brain. *Bottom*, scatter plot showing protein turnover changes in the hippocampus of symptomatic (P285) *TgCRND8* mice (see supplemental Table S1). In total, 847 proteins were quantified, and the difference in turnover characteristics between proteins in diseased and healthy animals was plotted as described in Figure 1*B* (2–3 mice in each cohort). *B*, *left* bar chart showing the average hippocampal protein turnover of 847 proteins in *TgCRND8* and WT mice. An overall 18.0% increase in protein turnover was detected (*p* < 0.0001). Error bars indicate SEM. ∗∗∗*p* < 0.0001. *Right* bar chart showing average plasma protein turnover in *TgCRND8* and WT mice (2–3 mice in each cohort). No significant difference was detected (*p* = 0.102, n = 74). Error bars indicate SEM. ns, not significant. *C*, scatter plot showing protein turnover changes in the cortex of presymptomatic (P186) *App*^*NL-F/NL-F*^ mice (see supplemental Table S1). In total, 721 proteins were quantified, and the difference in turnover characteristics between proteins in diseased and healthy animals was plotted as described in Figure 1*B* (2–3 mice in each cohort). *D*, *left* bar chart showing the average cortex protein turnover of 721 proteins in *App*^*NL-F/NL-F*^ and WT mice (2–3 mice in each cohort). No change in protein turnover was detected (*p* < 0.0735). Error bars indicate SEM. ns, not significant. *Right* bar chart showing average plasma protein turnover in *App*^*NL-F/NL-F*^ and WT mice (2–3 mice in each cohort). No significant difference was detected (*p* = 0.534, n = 48). Error bars indicate SEM. ns, not significant. *E*, *top*, immunohistochemical detection of synaptic marker, *left*, Psd95 and *right*, β-amyloid pathology in sagittal sections of symptomatic *App*^*NL*-*F*/*NL*-*F*^ (P500) mouse brain. *Bottom*, scatter plot showing protein turnover changes in the cortex of symptomatic (P548) *App*^*NL-F/NL-F*^ mice from (see supplemental Table S1). In total, 721 proteins were quantified, and the difference in turnover characteristics between proteins in diseased and healthy animals was plotted as described in Figure 1*B* (2–3 mice in each cohort). *F*, *left* bar chart showing the average cortex protein turnover of 847 proteins in *App*^*NL-F/NL-F*^ and WT mice (2–3 mice in each cohort). An overall 15.7% increase in protein turnover was detected (*p* < 0.0001). Error bars indicate SEM. ∗∗∗*p* < 0.0001. *Right* bar chart showing average plasma protein turnover in *App*^*NL-F/NL-F*^ and WT mice (2–3 mice in each cohort). No significant difference was detected (*p* = 0.961, n = 95). Error bars indicate SEM. ns, not significant. *G*, scatter plot showing protein turnover changes in the spinal cord of acutely symptomatic (P120) *TgSOD1-G93A* mice (see supplemental Table S1). In total, 496 proteins were quantified, and the difference in turnover characteristics between proteins in diseased and healthy animals was plotted as described in Figure 1*B* (2–3 mice in each cohort). *H*, *left* bar chart showing the average protein turnover of 496 proteins in the spinal cord of *TgSOD1-G93A* and WT mice (2–3 mice in each cohort). An overall 17.6% increase in protein turnover was detected (*p* < 0.0001). Error bars indicate SEM. ∗∗∗*p* < 0.0001. *Right* bar chart showing average plasma protein turnover in *TgSOD1-G93A* and WT mice (2–3 mice in each cohort). No significant difference was detected (*p* = 0.417, n = 89). Error bars indicate SEM. ns, not significant.

**Fig. 3:**
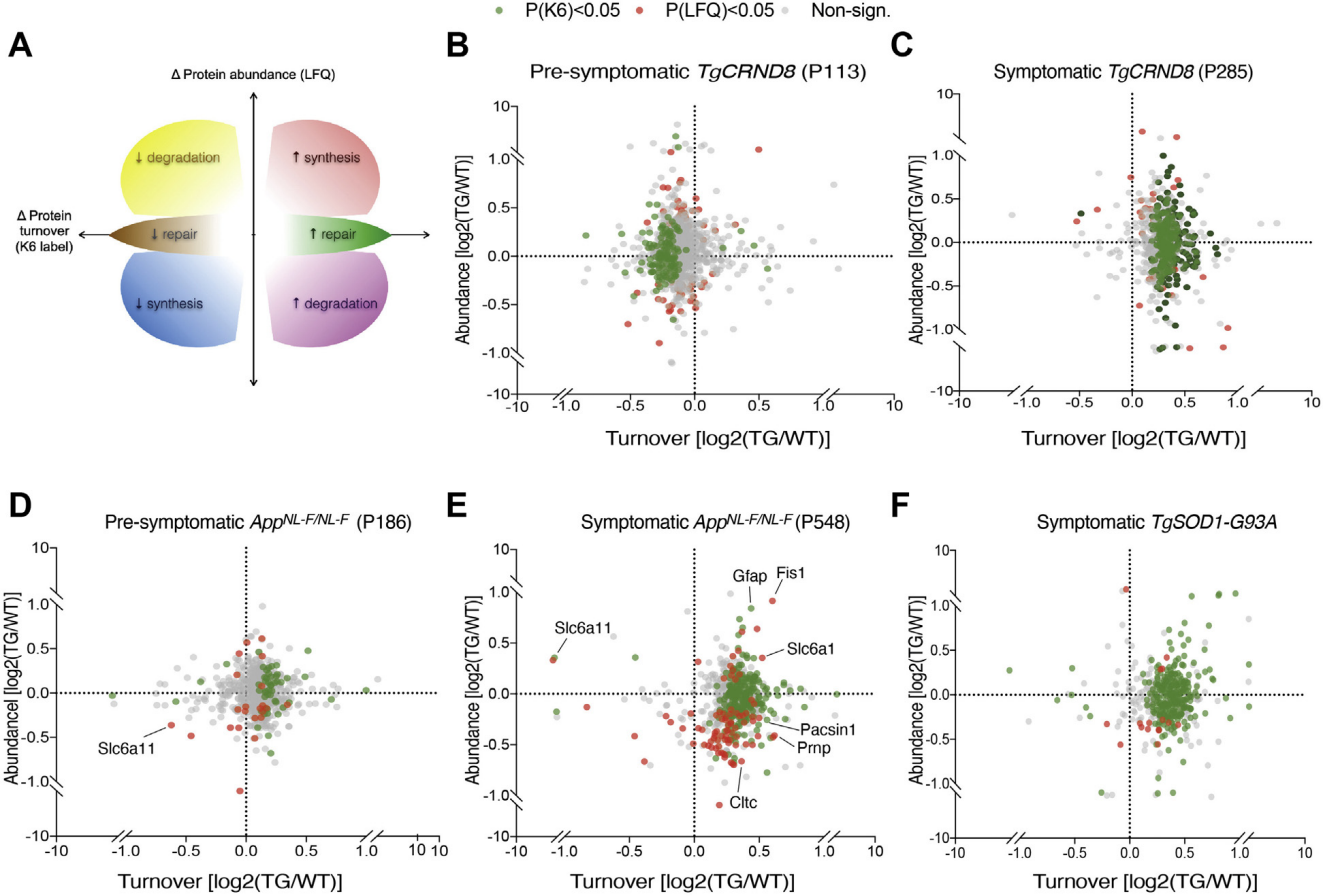
**Multidimensional measurement of proteome dynamics in live mice.***A*, schematic of a dynaplot showing the change in protein turnover (*x*-axis, K6 label) plotted against the change in protein abundance (*y*-axis, LFQ). The coordinate space of the plot reflect dynamics of a protein that can be attributed to a net increase (*red*) or decrease (*blue*) in the rate of synthesis; net increase (*magenta*) or decrease (*yellow*) in degradation. Change in turnover that does not result in a change in steady-state expression indicate change in flux of increasing (*green*) or decreasing repair (*brown*). *B*, dynaplot of hippocampal proteins in presymptomatic (P113) *TgCRND8* mice, as compared with healthy, matched control mice (2–3 mice in each cohort). Proteins that were significantly different in turnover (*p* < 0.01) are highlighted in *green*, while those that were significantly different in steady-state amount (*p* < 0.01) are highlighted in *red*. *C*, dynaplot of hippocampal proteins in acutely symptomatic (P285) *TgCRND8* mice, as compared with healthy, matched control mice. *D*, dynaplot of cortex proteins in presymptomatic (P186) *App*^*NL-F/NL-F*^ mice, as compared with healthy, matched control mice. *E*, dynaplot of cortex proteins in symptomatic (P548) *App*^*NL-F/NL-F*^ mice, as compared with healthy, matched control mice. *F*, dynaplot of hippocampal proteins in acutely symptomatic (P120) *TgSOD1-G93A* mice, as compared with healthy, matched control mice. See supplemental Table S4.

**Fig. 4:**
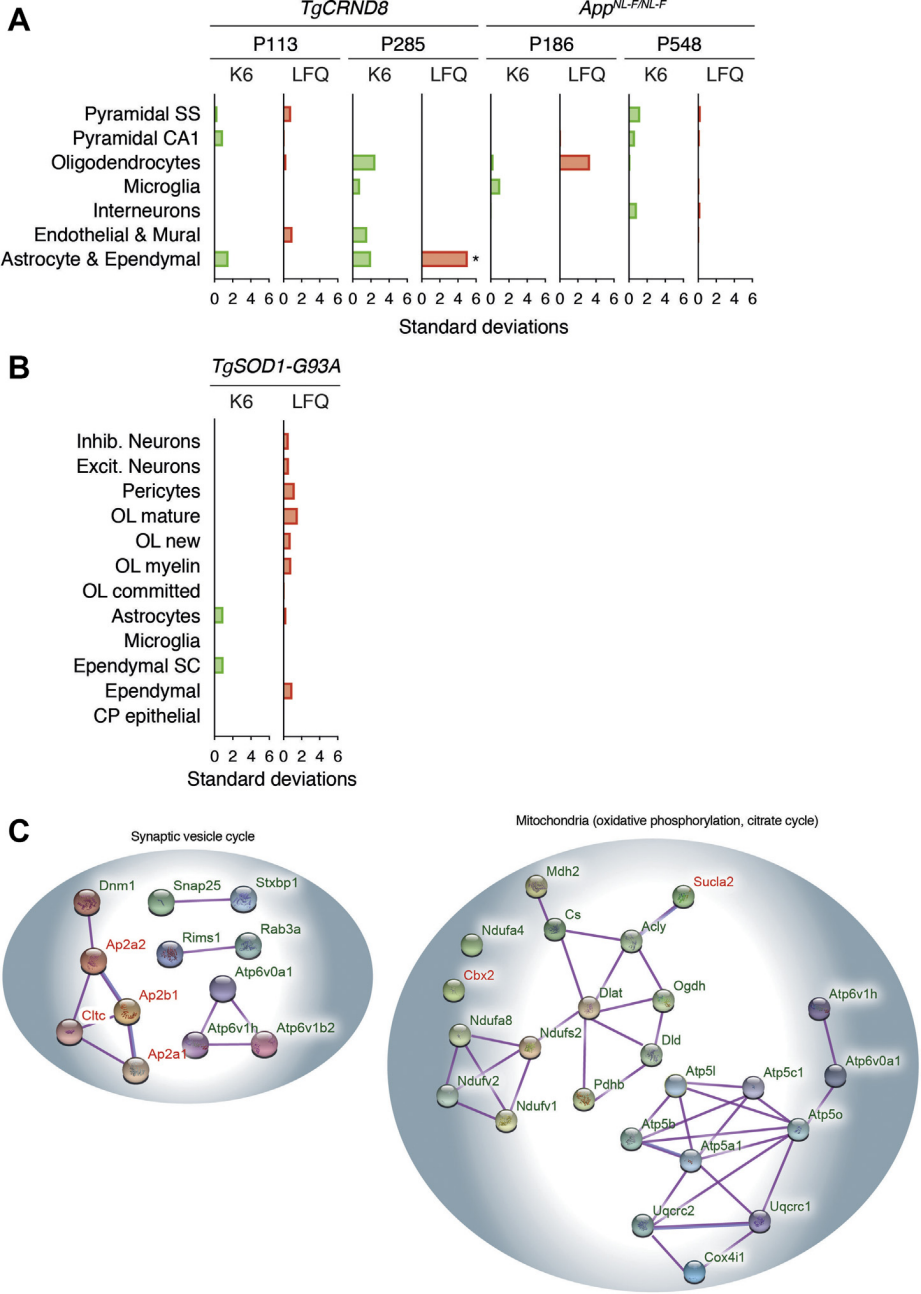
**Cell type enrichment and proteins with perturbed dynamics converge on presynaptic functions in mouse model of Alzheimer’s disease.***A*, cell type enrichment of the top 1% of differentially expressed genes from each experiment (P113 and P285 *TgCRND8*; P186 and P548 *APP*^*NL-F/NL-F*^) for both protein turnover (K6) and label-free protein quantification levels (LFQ). Cell type enrichment was produced using Expression Weighted Cell Type Enrichment (EWCE) with bootstrap sampling repeated 10,000 times. *y*-axis, cell type. *x*-axis, standard deviations from the mean specificity in that cell type. ∗, corrected *p* < 0.05 (Benjamini and Hochberg). *B*, same as *A* but for *TgSOD1-G93A* using spinal cord EWCE dataset. Inhib. and Excit., spinal cord inhibitory and excitatory neurons, respectively. OL, oligodendrocytes. Ependymal SC, spinal cord ependymal cells. CP epithelial, chorid plexus epithelial cells. *C*, gene ontology enrichment using KEGG database (see Experimental Procedures) identified *left*, synaptic vesicle recycling, and *right*, mitochondrial pathways enriched with the proteins whose turnover (*green*) or steady-state amount (*red*) has significantly changed in symptomatic (P548) *App*^*NL-F/NL-F*^ cortex. *Purple* and *blue* edges indicate empirically determined protein–protein interactions and protein homology, respectively.

**Fig. 5:**
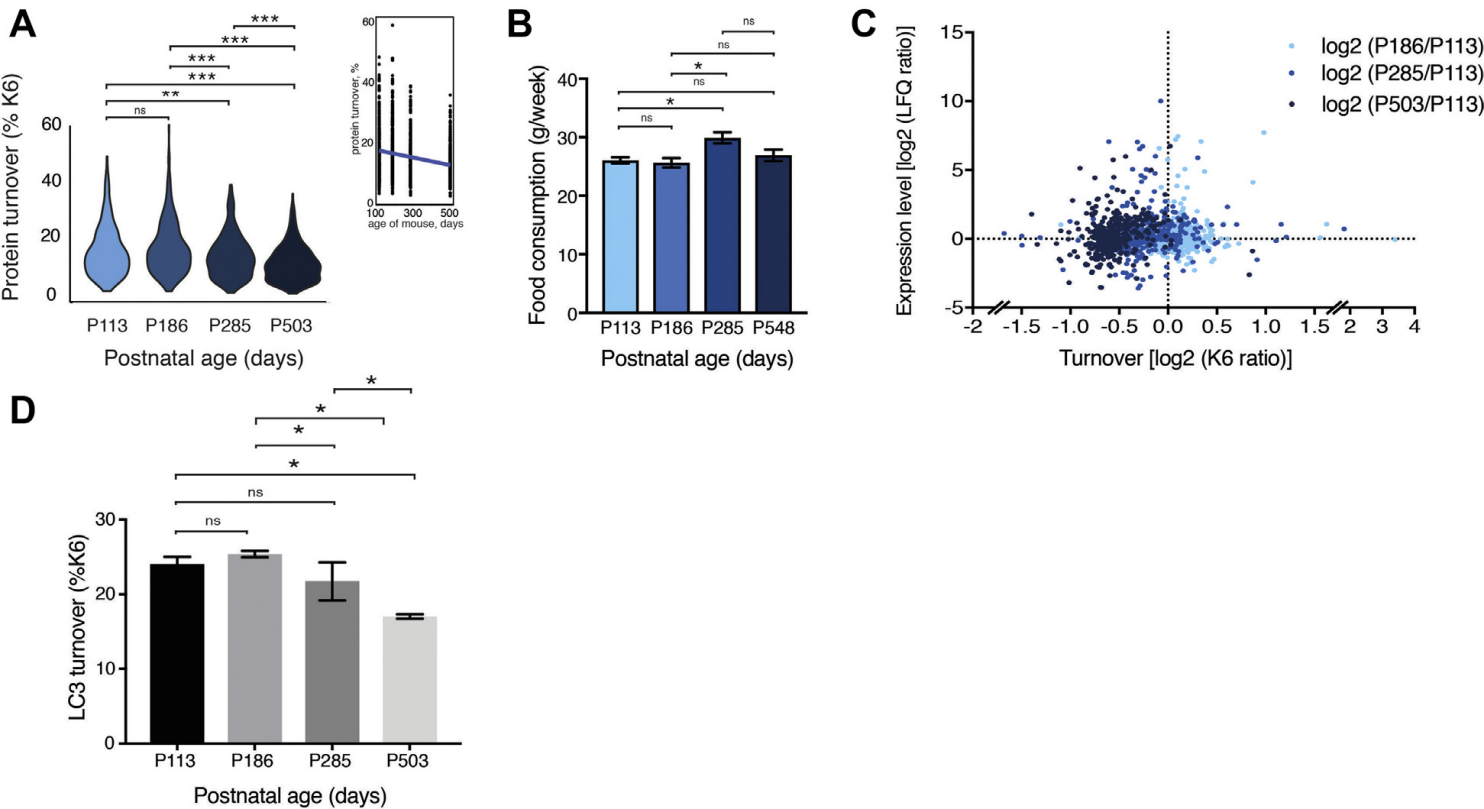
**Proteome dynamics associated with aging in healthy mice.***A*, average protein turnover in healthy mouse cortex at various ages. In total, 360 proteins were used to compare turnover across all ages (n = 3 at each postnatal age). *p* values for the comparisons (*descending from top*): 1.063e^−06^, 4.557e^−14^, 1.220e^−05^, 6.799e^−14^, 0.003, 0.175. *B*, average food consumption of mice at different ages: P113 (n = 4), P186 (n = 2), P285 (n = 4), P548 (n = 11). No significant change in appetite associated with aging was detected. *p* values for the comparisons (*descending from top*) = 0.127, 0.675, 0.047, 0.694, 0.011, 0.692. *C*, dynaplot of mouse cortex proteins depicting the change in proteome dynamics in healthy control mice from P113 to P186 (*sky blue*), to P285 (*blue*), and to P548 (*dark blue*). These data are expressed ratiometrically to allow simpler visualization between different ages of mice. *D*, bar graph showing apparent decrease in turnover of LC3, marker of autophagy, as mice age. Turnover was estimated by percentage heavy lysine incorporation (P6) in mice at postnatal days 113, 186, 285, and 503. ∗*p* < 0.05. n.s., non-significant.

At the end of the labeling period, the mice were culled and all major organs were collected and immediately frozen in liquid nitrogen, including blood plasma and cerebrospinal fluid. The brain was dissected into eight separate regions, and each area was frozen individually and immediately (olfactory bulb, caudate putamen, hippocampus, cortex, colliculus, cerebellum, thalamus, and hindbrain).

### Mouse Tissue Fractionation

Mice were culled by cervical dislocation or by overdose of pentobarbital. Organs were immediately dissected on ice, including the separation of brain areas and harvesting of cerebrospinal fluid, and all tissue and humors were snap frozen in liquid nitrogen. Tissue was homogenized manually in H buffer (320 mM sucrose, 2 mM HEPES at pH 7.3 with protease inhibitors). Volumes of H buffer were scaled to mass of tissue (232 mg tissue = 5 ml H buffer). Nuclei were pelleted by centrifugation at 1000*g*, and this pellet was homogenized for a second time in H buffer and pelleted as above. Membranes were pelleted from the supernatant at 21,000*g*, and all fractions were divided into small portions and flash-frozen in liquid nitrogen. This procedure was applied to the hippocampus, cortex, and spinal cord. Plasma proteins were diluted 1:40 with PBS and added to LDS sample buffer (Thermo Fisher) directly. For extraction, tissue fraction pellets were resuspended in H buffer, and extraction/precipitation buffer added to the suspension as appropriate. Extraction/precipitation buffer consisted of 25 mM Tris, pH 8, 50 mM NaCl, 2 mM TCEP, protease inhibitors, benzonase (Novagen), and detergent—proteins from the membranes were solubilized in deoxycholate or Triton X-100 (0.8% w/v or 1% v/v final, respectively), and proteins from the nuclear fraction were precipitated in Triton X-100 or GDN (1% final). Solubilized material was cleared by ultracentrifugation at 12,000*g* for 40 min, 8C, and precipitated material was pelleted by centrifugation at 21,000*g* for 25 min, 8C. Solubilized or precipitated material was prepared for SDS-PAGE by addition of LDS sample buffer, and cysteines were alkylated with 10 mM iodoacetamide prior to electrophoresis through 1 mm thick 4 to 12% Bis-Tris acrylamide gels (Thermo Fisher). Proteins were stained with colloidal Coomassie blue, and 16 sections, each 4 × 4 mm, were cut from each sample lane and diced into 1 mm cubes separately. These polyacrylamide cubes containing the fractionated proteins were prepared for mass spectrometric analysis using the Janus liquid handling system (PerkinElmer). Briefly, the excised protein gel pieces were placed in a well of a 96-well microtitre plate and destained with 50% v/v acetonitrile and 50 mM ammonium bicarbonate, reduced with 10 mM DTT, and postalkylated with 55 mM iodoacetamide. After alkylation, proteins were digested with 6 ng/μl trypsin (Promega) overnight at 37 °C. The resulting peptides were extracted in 2% v/v formic acid, 2% v/v acetonitrile. In some cases, the peptides extracted from 16 gel sections were combined into four samples for LC-MS. The conditions of detergent fractionations and digestion with trypsin were chosen from several rounds of optimization experiments to maximize peptide coverage and quantification.

### Mass Spectrometry and Data Analysis

The protein digest was analyzed by nano-scale capillary LC-MS/MS using an Ultimate U3000 HPLC (Thermo Fisher Dionex) to deliver a flow of approximately 300 nl/min. A C18 Acclaim PepMap100 5 μm, 100 μm × 20 mm nanoViper (Thermo Fisher Dionex), trapped the peptides prior to separation on a C18 Acclaim PepMap100 3 μm, 75 μm × 250 mm nanoViper (Thermo Fisher Dionex). Peptides were eluted with a 120 min gradient of acetonitrile (2%–50%). The analytical column outlet was directly interfaced, *via* a nano-flow electrospray ionization source, with a hybrid quadrupole orbitrap mass spectrometer (Q-Exactive Plus Orbitrap, Thermo Fisher). Data-dependent analysis was carried out, using a resolution of 30,000 Da for the full MS spectrum, followed by ten MS/MS spectra. MS spectra were collected over an m/z range of 300 to 2000. MS/MS scans were collected using a threshold energy of 27 for higher-energy collisional dissociation (HCD). Each tryptic peptide containing lysine-6 produced a peptide ion pair differing by 6.02 Da (divided by charge state).

For SILAM analysis of protein turnover, peptide pairs were located with MaxQuant 1.5.0 and identified with Andromeda using a reviewed version of the mouse Uniprot database (release_2013_01, 17082 forwarded entries) (40), allowing two missed cleavages at Lysine/Arginine. Cysteine carbamidomethylation was set as a fixed modification and *N*-acetylation of protein and oxidation of methionine as variable modifications. The mass tolerance was set to ± 20 ppm for MS and ± 0.5 Da for MS/M, and data were filtered with a 1% FDR at peptide and protein level. Each detergent fraction was analyzed with MaxQuant individually, and quantified proteins were forwarded for analysis if they were found in at least two of three biological replicates in both control and experimental animals. A final nonredundant merged dataset was generated excluding quantifications of the same protein from different detergent fractions; keeping the protein measurement with the greatest difference in Lysine-6 incorporation between the control and experimental animals. Two-tailed Student’s *t* tests (*p* = 0.05) were performed on the ratios of incorporation in control and experimental animals. As detailed in Figure 1*A*, the SILAM ratio is a direct readout of protein turnover.

For Label-Free Quantification of the same datasets, the maxLFQ functionality of MaxQuant 1.5.0 was used (41). However, proteins in each detergent fraction were forwarded for subsequent analysis only if they were found in every single mouse of that fraction (six of six). The protein quantification data were integrated by averaging the quantities from each high-quality detergent fraction together on a mouse-by-mouse basis. Two-tailed Student’s *t* tests (*p* = 0.05) were performed on the levels of protein in control and experimental animals.

Importantly, it was essential to observe the peptide pairs at least twice, by using a double count requirement for quantification in MaxQuant. We found experimentally that if the pairs of peptides were measured only in a single scan, peptide ratios were erroneously found in over 2% of identified proteins in an unlabeled sample (57 ratios in 2733 identifications, data not shown).

### Immunohistochemistry

Mice were euthanized by cervical dislocation or by overdose of pentobarbital when they were transcardially perfused with PBS. Brains were divided along the midline, and half was submerged in OCT (optimal cryotomy) solution in a cut-away plastic mold—the other half was kept for biochemical analysis. OCT-submerged brains were frozen by submersion of the mould into a beaker of isopentane that was subsequently chilled in liquid nitrogen. The frozen brain sections were cut at a thickness of 14 um using a cryostat (Leica) and postfixed using freezing methanol. Sections were blocked using 3% BSA or 10% goat serum in PBS with 0.2% Triton X-100 and probed with primary antibodies [Mouse anti-Abeta (6E10; Covance, 803001) and Rabbit anti-PSD95 (Abcam, ab18258] overnight at 4 °C. Secondary antibodies were conjugated to Alexafluor 488 or 647 (Thermo Fisher) and were applied to the samples for 2 h before mounting the slide using ProLong Antifade mountant with DAPI (Thermo Fisher). Images were acquired using a Zeiss LSM780 confocal microscope, then viewed and analyzed in Fiji. All antibody combinations were validated by controls with individually absent primary antibodies.

### Immunoaffinity Protein Purification

Frozen membrane fractions were resuspended in H buffer and solubilized in DOC extraction buffer (as above) for 1 h. The extract was cleared by ultracentrifugation (120k x g, 40 min, 8C). Antibodies were added to the cleared extract and left to bind overnight at 4C. The antibodies and adsorbed proteins were reclaimed by the addition of protein G Dynabeads (Sigma) for 40 min the following day. After washing twice with a solution containing 25 mM Tris, pH 8, and 50 mM NaCl, the antibodies and the adsorbed proteins were eluted with SDS.

### Expression Weighted Cell Type Enrichment (EWCE) Analysis

Differentially expressed genes (DEGs) were first derived using limma (42). Between-array quantile normalization was performed using the voom function. The gene lists from the experiments were then filtered to remove genes with multiple MGI symbols and genes, which were not present in the EWCE reference dataset (supplemental Table S6). EWCE used single-cell RNA sequencing (scRNA-Seq) dataset as a reference to derive cell type enrichments based on the inputted DEGs. The origin of scRNA-Seq datasets matched that of the proteomic data: cortex/hippocampus (43) and spinal cord (44) for β-amyloidosis (*TgCRND8* and *App*^*NL-F/NL-F*^) and TgSOD1-G93A proteomic datasets, respectively. For each experiment, EWCE's analysis was conducted on the top 1% of up- and downregulated DEGs, using bootstrap sampling repeated 10,000 times and a Benjamini and Hochberg adjusted *p* value threshold of 0.05.

### Functional Clustering Analysis

Proteins that changed their turnover dynamics were functionally clustered by the DAVID online tool (https://david.abcc.ncifcrf.gov/home.jsp), using KEGG pathways. Functional annotation charts were exported and visualized using String (45) to depict experimentally determined protein–protein interactions.

## Results

### Detecting Proteome Turnover in Mouse Models of Disease

There are multiple approaches capable of measuring protein turnover (46, 47, 48, 49). We devised a simple approach that matched the following three criteria: (1) requires minimal experimental design, (2) is amenable to cohorts of multiple test and control mice, and (3) enables straightforward identification of peptides, label incorporation, and turnover. To quantify changes in protein turnover, mice were fed a diet in which the essential amino acid, lysine (K0), was replaced with a ^13^C stable isotope derivative (K6) for 6 to 8 days (Fig. 1*A*). The rate of K6 incorporation was directly quantified by the ratio of K6 to K0 in each mouse (supplemental Fig. S1*A*). We benchmarked the accuracy of identifying heavy–light peptide pairs using a decoy unlabeled dataset, which indicated an empirical FDR of 2% and 0.01% using single and double counting of K0-K6 pairs, respectively. Therefore, double counts were used throughout.

To validate this simple method of measuring changes in protein turnover and its applicability to neurodegenerative disease, we used *TgCRND8* mice, an aggressive transgenic mouse model of familial AD that overexpresses hAPP (37). This mouse line develops several pathologies characteristic of AD including β-amyloid plaques, synapse loss, and behavioral phenotypes (37). Three 3-month-old *TgCRND8* and three age-, sex-, and genetic background-matched control mice were labeled with K6 food for 6 days. At the end of this labeling period, the hippocampi from these mice were collected for LC-MS (Fig. 1*A*). Measuring the change in K6 incorporation between disease and control tissues gave a snapshot of proteome turnover associated with the disease.

In each tissue sample from each mouse, we identified an average of 72,261 peptides (±4231 sd), with a total of 130,516 unique peptides identified in the cohort. Of this total, 67,416 peptides contained at least a single lysine residue, and we identified light–heavy (K0-K6) labeled peptide pairs in 41.25% of them, enabling the quantification of label incorporation in 27,752 peptides (supplemental Table S1). Overall, these data gave rise to 10,973 (±392 sd) identified proteins, and K6 incorporation was quantified in 2685 (±190 sd) proteins per tissue sample. The change in turnover was calculated using proteins detected in at least two mice from each cohort, giving a screen that measured 1392 protein turnover changes in the *TgCRND8* hippocampus with high-quality MS data. A few proteins were absent from the screen because they contain very few lysine-containing peptides. One example of these proteins, ApoE, was of particular interest because of its association with AD. Therefore, to quantify these extremely scarce ApoE peptides, we enriched samples by immunoaffinity purification before MS analysis (supplemental Fig. S1*B*). In principle therefore, this *in vivo* approach can detect changes in protein turnover of any lysine-containing protein in any tissue.

Incorporation of K6 ranged from 1.4 to 70.9%, indicative of a large dynamic range of protein turnover. Remarkably, although there is minimal β-amyloid pathology at this age (Fig. 1*B* and supplemental Table S1), an overall 6.1% slowdown in the global average protein turnover (GAPT) was measured in *TgCRND8* hippocampus (*p* = 0.0037 n = 1392, Fig. 1*C*). In contrast, serum (*p* = 0.128, n = 118) and liver (*p* = 0.17, n = 590) protein turnover did not change significantly (Fig. 1, *D* and *E*), indicating that the decrease in protein turnover is specific to the pathologically affected forebrain tissue. As a further control to account for amino acid recycling rates, the precursor K6 concentration was determined using peptides containing more than one lysine in disease and control samples (46, 50). No difference was detected (supplemental Table S2), indicating that changes in K6 incorporation directly measure changes in protein turnover.

If turnover changes are associated with β-amyloidosis, then as pathology progresses, one would expect protein turnover changes to reflect the increasing load of β-amyloid pathology. To test this possibility, we repeated our turnover measurement at P285, by which age *TgCRND8* has pervasive amyloid deposits throughout the forebrain (Fig. 2*A*, *top*, and supplemental Table S1). Surprisingly, examination of the protein turnover in the older *TgCRND8* model did not extend the slowdown that was seen at 113 days of age. Instead, at 285 days of age, an 18% increase in hippocampal GAPT (*p* < 0.0001, n = 847, Fig. 2, *A* and *B*), whereas in serum protein GAPT was unchanged, indicating that the change in proteome flux was restricted to the locus of β-amyloidosis. Overall, these proteome turnover data indicate discordance in proteome kinetics between early and late stages of pathology in the *TgCRND8* model of AD.

### Proteome Turnover in the *App*^*NL/F*^ Knockin Mouse Model of AD

In progressive diseases, identifying protein turnover changes that precede pathology could indicate upstream molecular pathways involved in the disease. However, in transgenic models of disease, including *TgCRND8*, one cannot distinguish between *bone fide* pathological mechanisms and effects that result from ectopic overexpression of the APP precursor. Therefore, to test in the absence of overexpression, we used a knockin mouse model of familial AD, *App*^*NL-F/NL-F*^, which lacks these potential artifacts (38). Protein turnover was measured at P186 and P548, which are time points before and after widespread β-amyloid pathology, respectively.

In presymptomatic P186 *App*^*NL-F/NL-F*^ forebrain, no significant change in GAPT was detected (Fig. 2, *C* and *D*). However, in P548 *App*^*NL-F/NL-F*^ mice with advanced β-amyloidosis, forebrain GAPT increased by 15.7% (*p* < 0.0001, n = 721), with 53 proteins being made or degraded faster (Fig. 2*E* and supplemental Table S1). No significant change was detected in serum proteins (Fig. 2*F*). Thus, an increase in protein flux is associated with increasing pathology in the *App*^*NL-F/NL-F*^ knockin mouse model of β-amyloidosis.

### Protein Turnover in Tissue Undergoing Cell Death

Late stages of neurodegenerative diseases are characterized by widespread neuronal death. To test for proteome turnover changes in tissues undergoing neuronal death, we K6-labeled 3-month-old *TgSOD1-G93A* mice (39), a mouse model of familial amyotrophic lateral sclerosis (ALS). At this age, *TgSOD1-G93A* mice displayed rear gait phenotypes, indicating spinal cord pathology and extensive neurodegeneration. In *TgSOD1-G93A* spinal cord GAPT increased 17.7% in the diseased mice compared with control (*p* = 0.0001, n = 496, Fig. 2, *G* and *H* and supplemental Table S1). In contrast, plasma protein GAPT was unchanged (*p* = 0.417, n = 89). Overall, in all models at late stages, a marked increase in overall protein flux was detected. These data could give insight into the particular pathways. However, this raises the question whether or not increased turnover is coupled to the gain or loss in the abundance of proteins, or if the increased turnover corresponds to the futile cycles of increased repair.

### Dynaplot: A Comprehensive Map of Proteome Dynamics

The kinetics of protein turnover drives the abundance of all proteins (11). Therefore, the simultaneous measurement of turnover and abundance of each protein can give a comprehensive description of proteome dynamics and mechanistic insight. Having established a method for screening changes in protein turnover, we next combined turnover measurements with relative abundance measurements by exploiting recent improvements of label-free quantification (MaxLFQ) (41). In each mouse model of disease dataset, an average of 4357 proteins (±654 sd) were quantified by label-free quantification (supplemental Fig. S2 and supplemental Table S3). Proteins were quantified in all mice in 98.0% (±2.0%) of the proteins that were used for turnover analysis, giving excellent reproducibility.

Plotting turnover *versus* abundance changes (hereon referred to as a dynaplot) is potentially a powerful tool because the coordinate space of these measurements infers the mechanism of change, as depicted in Figure 3*A*. In principle, changes in turnover can be regulated by either the rate of synthesis or degradation. Therefore, six scenarios arise: (1) increased steady state levels driven by an increase in protein turnover indicates the net synthesis rate has increased. (2) Increased steady state levels can also be driven by a net decrease in turnover, which reflects a decrease in the rate of protein degradation. Similarly, two distinct mechanisms for decreasing the steady state level of proteins can be directly inferred: (3) decreased steady state levels driven by a decrease protein turnover, which is the result of a net decrease in protein synthesis, and (4) decreased steady-state levels driven by an net increase in protein turnover, which is driven by a net increase in degradation. Finally, proteins can occupy coordinate space on the dynaplot in which (5) increases or (6) decreases in protein turnover are uncoupled from steady-state changes. These futile cycles reflect an increase and decrease in the rate of protein repair.

A dynaplot showing the change in flux *versus* the change of abundance of each protein is depicted in Figure 3. In each disease mouse model imbalances in turnover resulted in changes in abundance of a subset of the proteome (Fig. 3, *B*–*F*). Comparing 6-month- and 18-month-old *App*^*NL-F/NL-F*^ showed a fivefold increase in the number of significantly changed proteins (Fig. 3, *D* and *E* and supplemental Table S3). Thus, increased pathology correlated with increased imbalances in the proteome.

Multiple cell types have been implied in the pathogenesis of AD, including neurons and astroglial cells (22). The latter are known to proliferate particularly early in mouse models of AD (51) and might contribute to the changes in turnover and abundance detected in our proteomic data. To examine if affected genes in each disease model converged on particular cell types, we used expression weighted cell type enrichment (EWCE) analysis (52). Only P285 *TgCRND8* showed significant enrichment in astrocytes and ependymal cells based on the label-free protein quantification levels (correct *p* < 0.05, Fig. 4, *A* and *B*), but only in older mice (P258). Interestingly, this enrichment may be related to the prion protein promoter used to drive ectopic expression of the transgene in *TgCRND8* (37), which is most active in astrocytes and epithelial cells (53). In contrast, the absence of significant enrichment of particular cell types in the *App*^*NL-F/NL-F*^ knockin mouse line, in which there is no overexpression of a transgene, is consistent with alterations in the proteome distributed among many cell types. However, we cannot exclude that our samples sizes were insufficiently powered to detect cell-type enrichments with effect size of protein turnover and abundance changes in animal models.

Since multiple different proteins were differentially affected, we explored if different pathways are impacted at early and late stages of pathology (supplemental Table S4). Analysis of the symptomatic *App*^*NL-F/NL-F*^ dataset using the KEGG showed that significantly changed proteins converged on several pathways that appear to be prevalent in presynaptic functions, including synaptic vesicle recycling and mitochondria (Fig. 4). Thus, multidimensional proteome dynamics have identified specific proteins and pathways dysregulated as a consequence of disease (supplemental Table S3).

### Proteome Dynamics of Aging and Autophagic Flux

Aging is the greatest known risk factor for developing neurodegenerative disease, including AD (54). It is commonly held that protein turnover slows with age (1, 55). Therefore, we measured the change in proteome dynamics associated with aging in wild-type mice varying in age from 3 to 17 months (Fig. 5*A* and supplemental Table S5). Strikingly, 98% of the proteins measured showed decreasing K6 incorporation with age (between 113 and 503 days of age, *p* = 6.2 x 10^−16^, n = 360). Thus, the global average protein turnover decreased significantly with age (Fig. 5*A*). Since the K6 label is delivered by diet, as a control we measured average food consumption, which indicated no decline in food consumption with age (Fig. 5*B*). The average levels of protein abundance did not change significantly (Fig. 5*C* and supplemental Table S5), consistent with earlier reports(56). Overall, these aging data could provide a rich resource for exploring molecular mechanisms associated with aging.

Next, we explored our proteome dynamics dataset to identify potential mechanisms that could explain the aging-associated global decrease in proteome turnover, focusing on mechanisms that could drive proteome wide changes. One such candidate is autophagy, the dynamics of which are challenging to follow (56, 57). A prominent marker of autophagy is LC3, and this showed a significant decrease in K6 incorporation that was tightly associated with aging (Fig. 5*D*). However, the steady-state levels of LC3 fluctuated but did not correlate with increasing age (supplemental Table S5). Thus, autophagic flux decreases with age and provides a mechanism that in part explains the global decrease in proteome turnover.

## Discussion

Using a novel combination of live mouse labeling and proteomic profiling, we have developed a method for simultaneously measuring the flux of proteome-wide changes in turnover and steady-state abundance. By combining both measurements, this approach distinguishes changes that are mediated by protein synthesis *versus* degradation and enables the direct estimate of changes in protein flux. We tested this method in multiple mouse models of disease, which revealed global effects as well as identifying individual pathways associated with pathology. Next, we applied the method to aging in wild-type mice and observed a decline in protein turnover that could be partially explained by a decline of *in vivo* autophagic flux.

This methodological framework should be especially useful for identifying proteins and molecular pathways in other live animal test settings, including disease models, learning, and behavior (58). Establishing the cause of an imbalance in turnover could be essential for understanding diseases and is likely to highlight new targets for pharmaceutical intervention. Indeed, there is a growing body of evidence that correcting imbalances in proteome dynamics can slow the onset of disease symptoms (59, 60).

There is also increased appreciation that better mouse models are needed to identify targets for therapeutic intervention in neurodegenerative diseases, including AD (61). The comprehensive proteome dynamics provided insights that enable the direct comparison of multiple different mouse models (62). Comparing the overexpressing hAPP transgenic model (*TgCRND8*) at early and late stages of the disease indicated large yet discordant effects on protein turnover. Since changes did not correlate with increasing pathology, it is difficult to distinguish molecular mechanisms altered by overexpression of the transgene from changes associated with β-amyloidosis in this mouse line. In contrast, turnover changes identified in models that do not rely on ectopic overexpression (*App*^*NL-F/NL-F*^ knockin model) were correlated with increased pathology.

In all models tested at symptomatic stages, global average protein turnover increased suggesting a disease-associated proteome-wide state of repair. Increased proteomic flux drove abundance imbalances caused by increased synthesis of one subset of the proteome and increased degradation of another (Fig. 3 and supplemental Table S4). This is consistent with transcriptomic data from AD postmortem samples that suggested increased autophagy-mediated turnover (63). It is likely transcriptional programs are involved in regulating the increase in proteome flux (64). Also, an increased flux of Aβ has been detected in familial AD patients (65).

In contrast to disease, it is intriguing that turnover declines as wild-type mice age, whereas steady-state levels of the proteome in mice appear to show no global change associated with age (66). This is consistent with similar reports of proteome turnover decline in invertebrates (67), albeit protein abundance in aging invertebrates appears to change (67, 68). Our turnover measurements of LC3 enabled an estimation of autophagic flux, which also declined with aging, suggesting that the global turnover decrease associated with aging could, at least in part, be mediated by a slowdown in autophagy.

Overall, it is striking that while aging in wild-type animals is associated with a decrease in flux, whereas aging in all three neurodegenerative disease models caused an increase in flux. Thus, these mouse models suggest that neurodegenerative diseases are not an acceleration of aging, but rather represent a state of proteome imbalance and increased repair. As improved models of neurodegenerative disease are developed, applying comprehensive proteome dynamics is expected to give important phenotypic, molecular, and mechanistic insight.

## Data Availability

All MS data were deposited in the PRIDE database (https://www.ebi.ac.uk/pride/archive/projects/PXD010671), project accession: PXD010671. The details of the deposited data are tabulated in supplemental Table S7.

## Supplemental data

This article contains supplemental data.

## Conflict of interest

The authors declare no competing interests.

## Supplemental Data

Supplemental Figures S1and S2
