# Supplementary material for: Multidimensional Dynamics of the Proteome in the Neurodegenerative and Aging Mammalian Brain
Source: Mol Cell Proteomics. 2021 Dec 31;21(2):100192. doi: 10.1016/j.mcpro.2021.100192 (PMC8816717; doi:10.1016/j.mcpro.2021.100192)
Supplement: Supplemental Figures S1and S2 [file mmc1.docx]

Supplementary information: **Multidimensional dynamics of the proteome in the neurodegenerative and aging mammalian brain**

**Authors**

Byron Andrews, Alan E. Murphy, Michele Stofella, Sarah Maslen, Leonardo Almeida-Souza, J Mark Skehel, Nathan G. Skene, Frank Sobott, René A. W. Frank

**Supplementary Methods**

| **REAGENT or RESOURCE** | **SOURCE** | **IDENTIFIER** |
| --- | --- | --- |
| **Antibodies** | | |
| Mouse monoclonal (4G8) anti-β-Amyloid (17-24) | BioLegend | Cat# 800701 AB_2313891 |
| Mouse monoclonal (6E10) anti-β-Amyloid (1-16) | BioLegend | Cat# 803015  AB_10102028 |
| Rabbit polyclonal anti-PSD95 | Abcam | Cat# ab18258  AB_ 444362 |
| Rabbit monoclonal (16H22L18) anti-ApoE | Thermo | Cat# 701241  AB_2532438 |
| Mouse monoclonal (D6E10) anti-ApoE | Abcam | Cat# ab1906  AB_302668 |
|  |  |  |
| **Deposited Data** | | |
| Raw and database-matched mass spec data | This paper | PRIDE: PXD010671 |
|  |  |  |
| **Experimental Models: Organisms/Strains** | | |
| *TgCRND8* | David Westaway | PMID: 11279122 |
| *App^NL-F^* | Takaomi Saido | PMID: 24728269 |
| *TgSOD1-G93A* | Jackson Labs | PMID: 8209258 |
|  |  |  |
| **Software and Algorithms** | | |
| MaxQuant 1.5.0 | Cox and Mann, 2008 | http://www.coxdocs.org/doku.php?id=maxquant:common:download_and_installation |
| Perseus | Tyanova *et al*, 2016 | http://www.coxdocs.org/doku.php?id=perseus:common:download_and_installation |
| Prism | Commercial software | https://www.graphpad.com/scientific-software/prism/ |
| R | Open source | https://www.r-project.org/ |


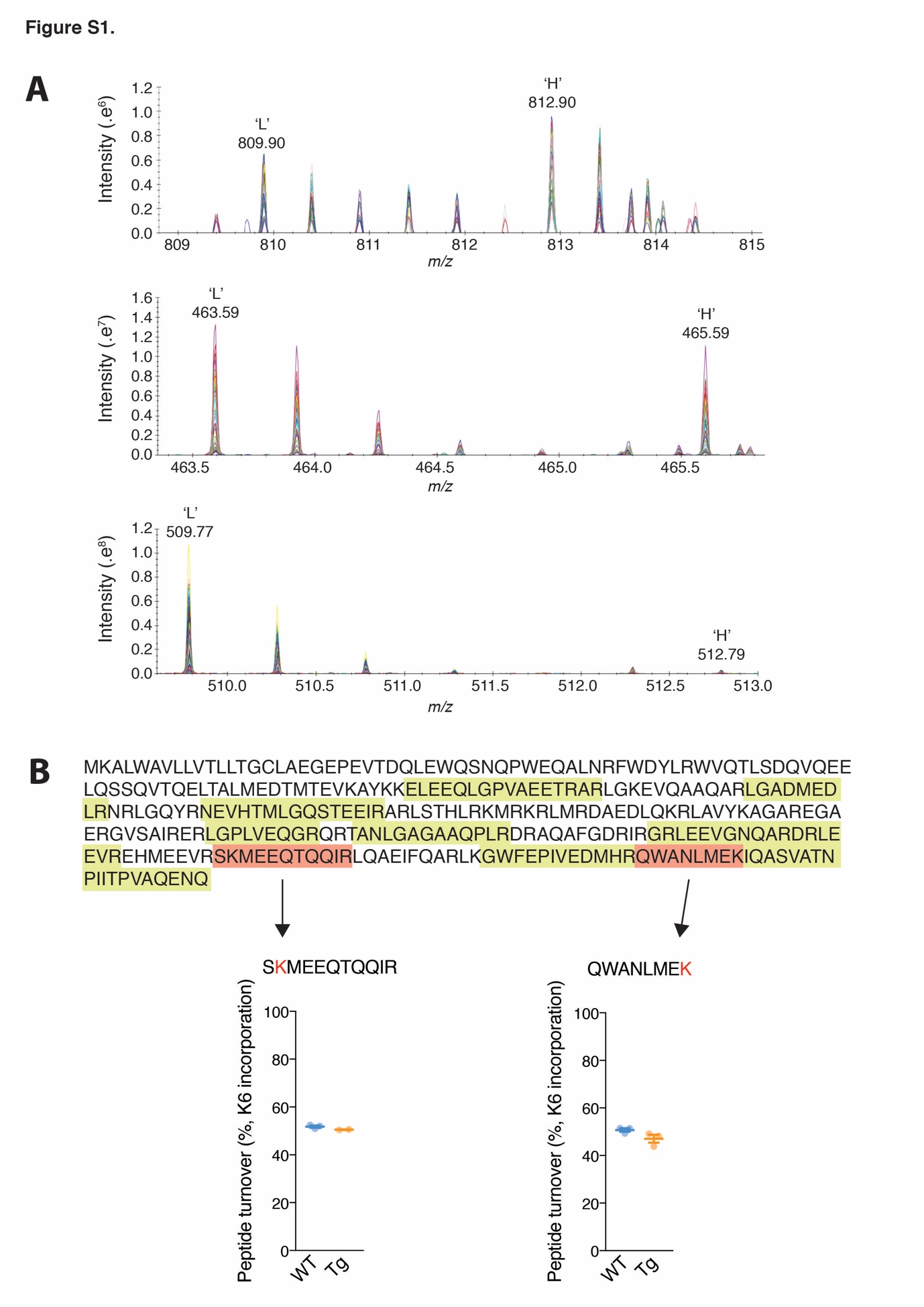


**Figure S1.**

**A** Representative examples of raw MS turnover measurements from P133 *TgCRND8* hippocampus showing heavy (‘H’, K6 labelled) and light (‘L’, unlabelled) peptide parent ions of Fbg (Fibrinogen beta), Slc25a13 (Calcium-binding mitochondrial carrier protein), Plp1 (myelin proteolipid protein) with high, medium and slow label incorporation, respectively. Spectra depicted as an overlay of multiple scans (each in a different color using SeeMs (Proteowizard).

**B** Mouse ApoE contains many tryptic peptides, but very few that contain lysine. To overcome this limitation for profiling protein turnover with heavy lysine (K6), we immuno-affinity purified ApoE from presymptomatic *TgCRND8* brain, and processed the purified protein for LC-MS. The 10 peptides that were consistently characterised are coloured, with the two Lysine peptides in red. No significant difference in ApoE turnover was apparent between the presymptomatic *TgCRND8* and healthy mice based on the K6 incorporation.

**
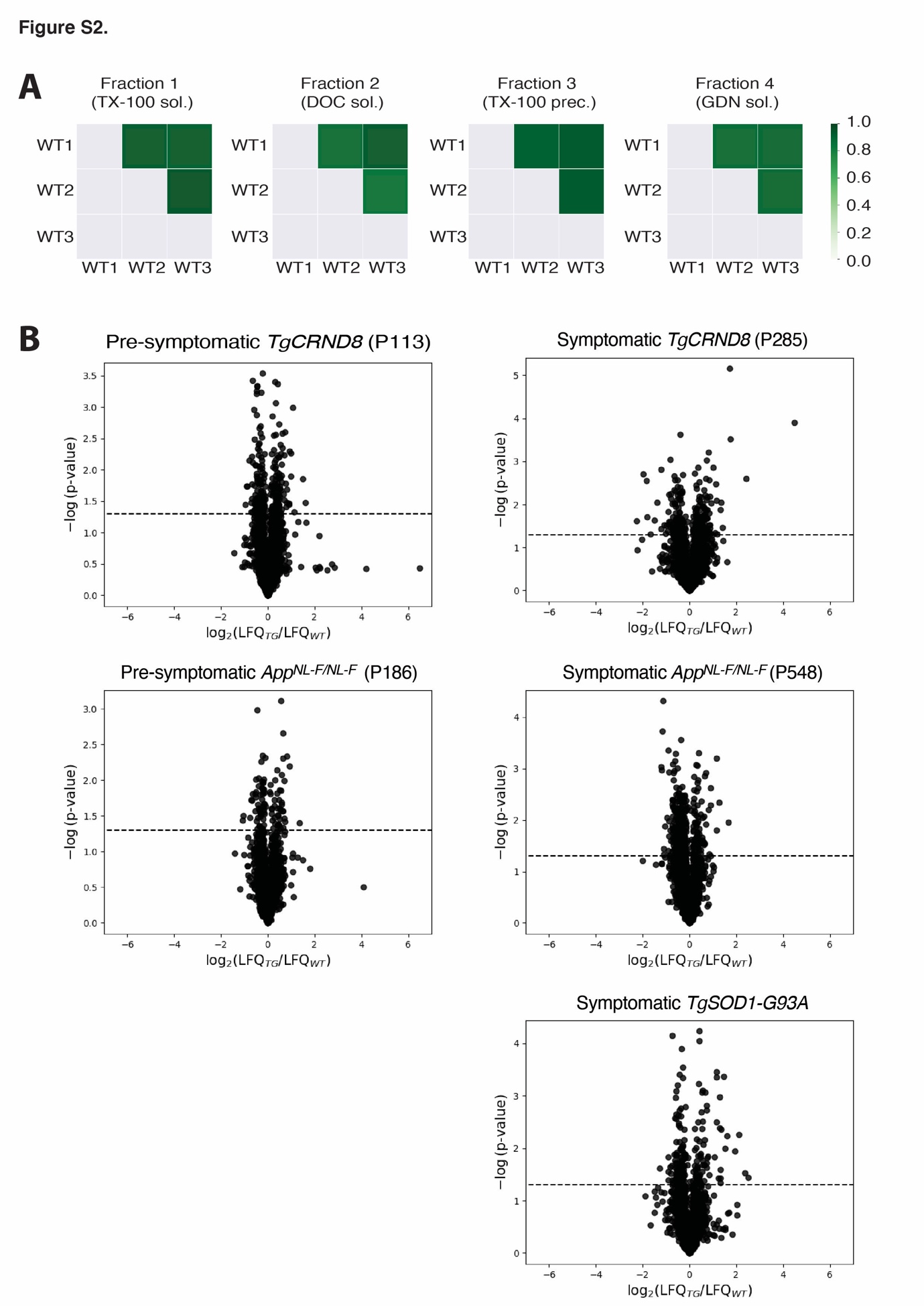
**

**Figure S2.**

**A** Heat maps showing the Pearson’s correlations of log_2_ transformed LFQ intensities to evaluate the consistency of raw spectra used for label free quantification in MaxQuant. Intensities of three WT mice were compared using spectra from four different detergent fractions from *left*, DOC, Triton X-100 supernatant solution, Triton X-100 precipitate, GDN supernatant solution, respectively. Pearson’s correlations ranged from 0.83-0.97. For all label-free quantification (LFQ), datasets used and passed MaxQuant normalisation.

**B** Volcano plots showing the differential protein abundance in five mouse models of neurodegenerative disease versus age-matched control (2 to 3 mice in each cohort). *Top left* and *right*, P113 and P285 *TgCRND8* hippocampus, respectively. *Middle left* and *right*, *APP^NL-F/NL-F^* cortex^­^. *Bottom right*, P120 *TgSOD1-G93A* spinal cord. *X-* and *y-*axis, log_2_ LFQ intensity and negative log_10_ P-value (uncorrected). Dashed horizontal line indicates P=0.05. These data, including protein identifies, LFQ measurements, and P-values, are tabulated in supplemental table S3.
